# Interfacial Assembly of Ti_3_C_2_T_x_/ZnIn_2_S_4_ Heterojunction for High‐Performance Photodetectors

**DOI:** 10.1002/advs.202204687

**Published:** 2022-10-26

**Authors:** Shuping Hou, Chen Xu, Xingkai Ju, Yongdong Jin

**Affiliations:** ^1^ State Key Laboratory of Electroanalytical Chemistry Changchun Institute of Applied Chemistry Chinese Academy of Sciences Changchun 130022 China; ^2^ School of Applied Chemistry and Engineering University of Science and Technology of China Hefei Anhui 230026 China

**Keywords:** interfacial assembly, liquid exfoliation, MXene, photodetector, two‐dimensional heterostructure, ZnIn_2_S_4_

## Abstract

Two‐dimensional (2D) materials have emerged as prospective candidates for electronics and optoelectronics applications as they can be easily fabricated through liquid exfoliation and used to fabricate various structures by further subsequent processing methods in addition to their extraordinary and unique optoelectronic properties. Herein, the Ti_3_C_2_T_x_/ZIS heterostructure with nanometer‐thick Ti_3_C_2_T_x_‐MXene and ZnIn_2_S_4_ (ZIS) films is fabricated by successive interfacial assembly of liquid exfoliated 2D MXene and ZnIn_2_S_4_ nanoflakes. Benefiting from the superior light‐harvesting capability and low dark current of ZnIn_2_S_4_, the limited absorbance, large scattering coefficient, and high dark current disadvantages of MXene are ameliorated. Meanwhile, the separation and transport of photogenerated carriers in ZnIn_2_S_4_ are improved due to the excellent electrical conductivity of Ti_3_C_2_T_x_ nanoflakes. As a result, the as‐prepared Ti_3_C_2_T_x_/ZIS heterostructure photodetector has excellent optoelectronic characteristics in terms of a high responsivity of 1.04 mA W^−1^, a large specific detectivity up to 1 × 10^11^ Jones, a huge on/off ratio at around 10^5^, and an ultralow dark current at ≈10^−12^ A. This work demonstrates a convenient method to construct heterostructured photodetectors by liquid exfoliated 2D nanoflakes, the as‐fabricated Ti_3_C_2_T_x_/ZIS heterostructured photodetectors show promising application potential for low‐cost, reliable, and high‐performance photodetectors.

## Graphical abstract

The interfacial assembly provides an emerging strategy to manipulate liquid‐phase exfoliated 2D materials into large‐scale and densely packed nanometer‐thick films for diverse optoelectronics applications. Benefiting from the good contact between the interfacial assembly nanoflakes and the synergistic interaction between MXene and ZnIn_2_S_4_ films, the as‐prepared Ti_3_C_2_T_x_/ZnIn_2_S_4_ photodetectors show excellent optoelectronic characteristics and potential for low‐cost, reliable, and high‐performance photodetectors.

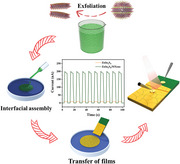

## 1 Introduction

Photodetector is an important class of optoelectronic devices that convert optic signals into electrical current which has widespread and pivotal applications in modern technologies, such as optical communication, optical imaging, biological detection, pollution monitoring, and so on.^[^ ^1^, ^2^, ^3^ ^]^ Among various photodetector materials, two‐dimensional (2D) materials, such as graphene, black phosphorus (BP), boron nitride, transition metal dichalcogenides (TMDs), transition‐metal carbides (MXene), etc., have been emerged as promising candidates for high‐performance photodetection applications, due to their extraordinary electrical, optical, physicochemical, and mechanical properties.^[^ ^4^, ^5^, ^6^, ^7^, ^8^ ^]^ Besides benefiting from the atomically thin planar structure, they can be combined into heterostructures with different materials by van der Waals (vdW) forces without considering the lattice mismatching condition, which expands the design strategy of 2D materials‐based optoelectronics devices beneficial for practical applications.^[^ ^4^, ^5^ ^]^ Numerous hybrid/heterojunction structures of 2D materials with 0D, 1D, 2D, or 3D materials have been envisioned as promising candidates for next‐generation photodetectors, which exhibit superior optoelectronic performances compared to single 2D materials.^[^ ^6^, ^7^, ^8^, ^9^, ^10^, ^11^, ^12^, ^13^ ^]^ Compared with the clean‐room fabrication methods like chemical vapor deposition (CVD), pulsed laser deposition (PLD), molecular beam epitaxial growth (MBE), etc., the wet‐chemical synthesis approaches provide an alternative route for low‐cost and large‐scale fabrication of 2D materials nanoflakes without the requirements of expensive instrumentation, stringent vacuum environments, and complex operations.^[^ ^14^, ^15^, ^16^, ^17^ ^]^ Due to weak inter‐layer bonds (van der Waals bonds) of the 2D materials, they can be easily separated into liquid‐dispersed mono‐ or few‐layered 2D materials nanoflakes by liquid exfoliation and used to construct 2D and 3D macrostructures and nanofilms by a range of subsequent processing methods such as layer‐by‐layer assembly (LBL), Langmuir–Blodgett assembly (LB), spin coating, inkjet printing, spray coating, vacuum filtration, interfacial assembly, freeze‐drying, and so on.^[^ ^18^ ^]^ Among these approaches, the interfacial assembly received enormous attention because it allows large‐scale production of densely packed nanometer‐thick films with good reproducibility and improved electrical conductivity, higher sensing factor, superior mechanical properties, and so on.^[^ ^19^ ^]^

MXene, as a new class of 2D transition metal carbides, nitrides, and carbonitrides, received widespread attention recently for diverse applications in electronics and optoelectronics, due to their intriguing optical and electrical properties and solution processability.^[^ ^20^, ^21^, ^22^, ^23^ ^]^ Benefiting from their intrinsically abundant terminal groups, MXene materials obtain tunable functional properties (electrical, optical, band gap, work function, etc.) and hydrophilic surfaces, which make them to be excellent interfacial assembly building blocks to create nanometer‐thick 2D nanofilms for diverse applications, such as transparent electrodes, electromagnetic interference shielding, electronic devices, sensor, and so on.^[^ ^19^, ^23^, ^24^, ^25^, ^26^, ^27^ ^]^

Among all MXene materials reported, Ti_3_C_2_T_x_ is the most representative and well‐studied. However, pure Ti_3_C_2_T_x_ materials exhibit almost no photocurrent response to the optical signal and extremely high dark current due to their limited absorbance, large scattering coefficient, and metallic conductivity, which severely hinders their practical applications in photodetectors.^[^ ^28^ ^]^ The combination of Ti_3_C_2_T_x_ with different semiconductors to form heterojunction is therefore an effective strategy to maximize the benefits of MXene and enhance the performance of photodetectors. So far, different MXene/semiconductor heterojunctions based on MXene/n‐Si, MXene/GaN, MXene/MoS_2_, MXene/TiO_2_, etc. in the field of optoelectronics have been extensively reported.^[^ ^24^, ^28^, ^29^, ^30^, ^31^, ^32^, ^33^, ^34^, ^35^ ^]^ ZnIn_2_S_4_ (ZIS) is a 2D layered ternary bi‐metal chalcogenide compound with a direct and adjustable band gap (1.72–2.48 eV) in the visible spectrum, superior light‐harvesting capability, favorable chemical stability, and environmental friendliness, which makes it extensively suitable for photocatalysis, photodetection, pollutant degradation, and fuel cells applications.^[^ ^36^ ^]^ However, the optoelectronic properties of ZnIn_2_S_4_ are unsatisfactory due to the short lifetime of photo‐induced charge carriers, which requires more efforts to further pursue higher optoelectronic performance of the systems.^[^ ^36^, ^37^, ^38^ ^]^ Based on the above considerations, the combination of MXene and ZnIn_2_S_4_ would promote optical absorption and response by the introduction of ZnIn_2_S_4_ while strengthen the separation and transport of photogenerated carriers with the benefit of the excellent electrical conductivity of Ti_3_C_2_T_x_ nanoflakes.^[^ ^39^, ^40^ ^]^

Herein, we succeeded in the fabrication of large‐scale nanometer‐thick MXene and ZnIn_2_S_4_ films by interfacial assembly of liquid exfoliated MXene and ZnIn_2_S_4_ nanoflakes at the water/*n*‐hexane interface, and further successively constructed them into multilayered heterostructured Ti_3_C_2_T_x_/ZIS photodetectors. Benefiting from the synergistic interaction between MXene and ZnIn_2_S_4_ heterostructure, the as‐prepared Ti_3_C_2_T_x_/ZIS heterostructure photodetector showed excellent optoelectronic characteristics including good responsivity (1.04 mA W^−1^), a superior detectivity (1 × 10^11^ Jones), a huge on/off ratio (≈10^5^), and a specific response time (646.8 ms) and recovery time (640 ms), which surpasses most reported photodetectors prepared by other liquid‐exfoliated 2D materials counterparts. The existence of ZnIn_2_S_4_ significantly enhances the overall light‐harvesting capability of the device leading to more carriers generation; whereas the superior electrical properties of MXene remarkably accelerate the kinetics of the separation and transport of photogenerated carriers. Our results suggest that the combination of MXene and liquid‐exfoliated 2D materials is an effective method to improve the performance of the corresponding photodetector. The Ti_3_C_2_T_x_/ZIS heterostructure photodetectors made by the interfacial assembly are promising for potential application as low‐cost, reliable, feasible and high‐performance photodetectors.

## 2 Results and Discussion

### 2.1 Characterizations of Ti_3_C_2_T*_x_* and ZnIn_2_S_4_ Nanoflakes

**Figure 1:**
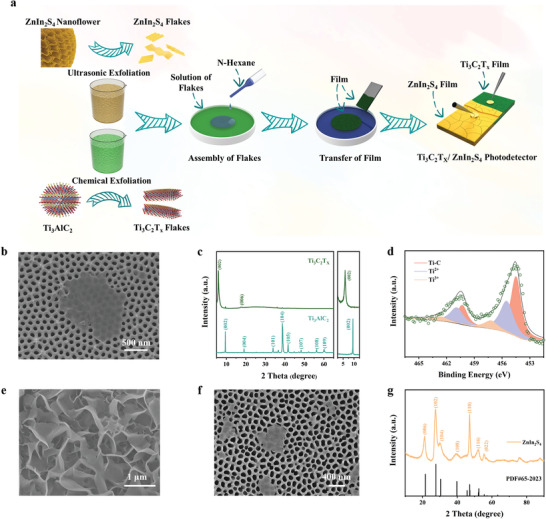
a) Schematic illustration of the interfacial assembly and transfer steps for fabricating the Ti_3_C_2_T_x_/ZIS based heterostructure photodetector. b) Scanning electron microscopy (SEM) image of the exfoliated monolayer Ti_3_C_2_T_x_ nanoflakes on AAO. c) X‐ray diffraction (XRD, left), and small‐angle X‐ray diffraction (SAXD, right) patterns of the Ti_3_AlC_2_ and Ti_3_C_2_T_x_. d) High‐resolution X‐ray photoelectron spectroscopy spectra of the Ti 2p region of Ti_3_C_2_T_x_ nanoflakes. e,f) SEM images of ZnIn_2_S_4_ nanoflowers and ZnIn_2_S_4_ nanoflakes, respectively. g) X‐ray diffraction pattern of the ZnIn_2_S_4_ nanoflakes.

The fabrication of the 2D Ti_3_C_2_T_x_/ZnIn_2_S_4_ based heterostructure photodetector by using the interfacial assembly approach is illustrated in **Figure** **1**a. Firstly, we synthesized both of Ti_3_C_2_T_x_ and ZnIn_2_S_4_ nanoflakes via a liquid exfoliation approach. 2D Ti_3_C_2_T_x_ nanoflakes were obtained by liquid chemical exfoliating MAX phase precursor (Ti_3_AlC_2_ powder) with a mixed solution of lithium fluoride and hydrochloric acid (details are provided in the Experimental Section, Supporting Information). Scanning electron microscopy (SEM, Figure 1b) and transmission electron microscopy (TEM, Figure S1a, Supporting Information) images revealed the typical ultrathin 2D layered structure of dispersed monolayers of Ti_3_C_2_T_x_ after the chemical exfoliation, which is significantly different from the MAX phase structures of Ti_3_AlC_2_ as shown in the SEM image (Figure S3a, Supporting Information), indicating that the 2D Ti_3_C_2_T_x_ nanoflakes were successfully prepared. More detailed crystallinity information of the MXene flakes was further obtained from high‐resolution TEM (HRTEM, Figure S2a, Supporting Information) images and the selected area electron diffraction (SAED, inset of Figure S2a, Supporting Information) patterns. The well‐defined lattice fringe with a distance of 0.31 nm is clearly observed, corresponding to the (110) planes of Ti_3_C_2_T_x_.^[^ ^41^ ^]^ The SAED pattern shows six folded symmetric diffraction spots, which indicates that Ti_3_C_2_T_x_ has a hexagonal atomic arrangement and good crystallographic characteristics in accordance with previous reports on Ti_3_C_2_T_x_ MXenes.^[^ ^42^ ^]^ Figure 1c shows the corresponding X‐ray diffraction (XRD) and small‐angle X‐ray diffraction (SAXD) patterns of the Ti_3_AlC_2_ raw powder before and after exfoliating. The observed (002) peak shifted to a smaller angle from 9.5° to 5.9° and the almost disappearance of characteristic peaks of Ti_3_AlC_2_ related to (101), (103), (104), and (105) indicated the increase of d‐spacing of the Ti_3_C_2_T_x_ nanoflakes from 9.3 to 14.6 Å after the treatment.^[^ ^43^ ^]^ The exfoliated Ti_3_C_2_T_x_ nanoflakes can be well‐dispersed in aqueous solutions, which displays the typical Tyndall effect as shown in the corresponding inset optical image of Figure S3b (Supporting Information), indicating the excellent hydrophilicity and dispersity of the Ti_3_C_2_T_x_ nanoflakes. We further characterized the optical characteristics of the Ti_3_C_2_T_x_ nanoflakes suspension using UV–vis absorption spectroscopy (Figure S3b, Supporting Information). The obvious absorption of the Ti_3_C_2_Tx nanoflakes suspension located at 760 nm as a result of the LSPR effect of the Ti_3_C_2_T_x_ nanoflakes was observed, which is in agreement with the previous reports.^[^ ^44^ ^]^ To obtain more information about the surface groups and chemical composition of the Ti_3_C_2_T_x_ nanoflakes, X‐ray photoelectron spectroscopy (XPS) was carried out. As shown in Figure S3c (Supporting Information), the significant signals from Ti, C, O, and F elements can be observed in the survey XPS spectrum of Ti_3_C_2_T_x_ where the peaks at binding energy values of 33, 101, 284, 453, 530, 682, and 985 eV corresponds to O 2s, Al 2s, C 1s, Ti 2p, O 1s, F 1s, and C KLL, respectively.^[^ ^45^, ^46^ ^]^ This result indicates that the surface of the as‐prepared Ti_3_C_2_T_x_ nanoflakes was functionalized mainly with –O groups, along with a few –OH and –F groups. Figure 1d shows the high‐resolution XPS spectra in the Ti 2p region, which contains three deconvoluted peaks corresponding to Ti–C, Ti^2+^, and Ti^3+^. It is believed that the Ti–C signal (454.6 and 460.3 eV) originates from Ti atoms in the internal layers of Ti_3_C_2_T_x_. As for the Ti signals with oxidation states, such as Ti^2+^ (455.54 and 460.9 eV) and Ti^3+^ (457.2 and 462.2 eV), these signals were possibly generated from the formation of mixed oxides (TiO_x_F_y_) and carboxyl groups (TiC_x_O_y_).^[^ ^45^ ^]^ And note that the Ti 2p XPS spectra showed no significant peak from Ti^4+^ of TiO_2_ at 458.8 eV, indicating no significant oxidation and degradation of the surface of Ti_3_C_2_T_x_. Therefore, the XPS analyses comprehensively elucidated the structural composition and surface functional groups (e.g., –F, –OH, and –Ox) of the Ti_3_C_2_T_x_ nanoflakes, of which the negative surface charge and hydrophilicity are critical for the subsequent fabrication of large‐scale nanometer‐thick Ti_3_C_2_T_x_ MXene films based on interfacial assembly strategy.

For the fabrication of ultrathin ZnIn_2_S_4_ nanoflakes, we prepared ZnIn_2_S_4_ nanoflowers via a facile low‐temperature refluxing method and then used an ultrasonic mechanical exfoliation method to exfoliate them into dispersed ZnIn_2_S_4_ nanoflakes (details are provided in the Experimental Section, Supporting Information). The SEM images of the ZnIn_2_S_4_ before and after the mechanical exfoliation are shown in Figure 1e,f, where the transformation of ZnIn_2_S_4_ from nanoflower‐like structures to typical 2D layer‐like structures can be observed obviously, demonstrating successful preparation of the ultra‐thin and transparent ZnIn_2_S_4_ nanoflakes. TEM image (Figure S1b, Supporting Information) further illustrates the ultrathin and transparent feature of the exfoliated product. The high‐resolution TEM image (Figure S2b, Supporting Information) exhibits a distinct lattice fringe with a distance of 0.32 nm, which corresponds to the (102) crystallographic plane of ZnIn_2_S_4_.^[^ ^40^ ^]^ The corresponding SAED pattern (inset of Figure S2b, Supporting Information) shows clear diffraction spots corresponding to the hexagonal structure of ZnIn_2_S_4_ nanosheets, which is further confirmed by XRD patterns. The XRD analysis (Figure 1g) revealed that the diffraction peaks of the as‐prepared ZnIn_2_S_4_ nanoflakes correspond well with the standard XRD patterns of the hexagonal of ZnIn_2_S_4_ (JCPDS No. 65‐2023), where the characteristic peaks of hexagonal ZnIn_2_S_4_ at 21.2°, 27.5°, 30.1°, 39.5°, 47.1°, 52.1°, and 55.5° correspond to the (006), (102), (104), (108), (110), (116), and (022) lattice planes, respectively. The valence band maximum (VBM) and conduction band minimum (CBM) of hexagonal ZnIn_2_S_4_ are positioned in the same k‐vector of the Brillouin zone, revealing its direct gap characteristics. This is attractive for the fabrication of high‐performance photodetectors because the direct band gap endows the semiconductor to have a greater light absorption capability.^[^ ^47^ ^]^ Subsequently, the XPS analysis was used to validate the surface elemental composition and the chemical state of the as‐prepared ZnIn_2_S_4_ nanoflakes. The survey XPS spectrum (Figure S4, Supporting Information) of the as‐prepared ZnIn_2_S_4_ nanoflakes reveals the existence of Zn, In, and S elements, while the signals of C and O come from the atmospheric adsorbates. The corresponding high‐resolution XPS spectra of the Zn 2p, In 3d, and S 2p regions are shown respectively in Figure S4b–d (Supporting Information). The peaks centered at 1021.6 and 1044.8 eV are attributed to the Zn 2p_3/2_ and Zn 2p_1/2_ states, respectively, while the peaks positioned at 444.8 and 452.4 eV are assigned to the In 3d_5/2_ and In 3d_3/2_ states. In addition, the high‐resolution XPS spectra of S 2p consists of two deconvoluted peaks where the peaks at binding energy values of 161.4 and 162.5 eV correspond to S 2p_3/2_ and S 2p_1/2_, respectively. These peaks observed are close to those of previous reports for ZnIn_2_S_4_, indicating the successful synthesis of ZnIn_2_S_4_ nanoflakes.^[^ ^37^ ^]^

### 2.2 Interfacial Assembly of MXene and ZnIn_2_S_4_ Nanoflakes

After obtaining aqueous Ti_3_C_2_T_x_ and ZnIn_2_S_4_ nanoflakes dispersions, the liquid/liquid interface assembly approach was used to fabricate large‐area few‐layered nanofilms of Ti_3_C_2_T_x_ and ZnIn_2_S_4_ nanoflakes. The optical images of the few‐layered ZnIn_2_S_4_ films and Ti_3_C_2_T_x_ films at the liquid/liquid interface are shown in Figures S5a,b (Supporting Information), respectively. Taking 2D Ti_3_C_2_T_x_ nanoflakes as an example, the typical process of interfacial assembly was further investigated and clarified in detail. In a typical preparation process as shown in Figure 1a, hexane was added to the aqueous solution of preformed 2D Ti_3_C_2_T_x_ nanoflakes to form a liquid–liquid interface. And a small amount of hydrochloric acid (HCl) was added to the solution to reduce electrostatic repulsion between individual Ti_3_C_2_T_x_ nanoflakes. Methanol was then injected as rapidly as possible into the two‐phase interface to induce compact monolayer assembly of the Ti_3_C_2_T_x_ nanoflakes at the liquid/liquid interface. With the continuous evaporation of hexane, a high‐surface tension gradient is established along with the liquid–liquid interface through the Marangoni force originating from the surface tension difference between water and hexane,^[^ ^26^ ^]^ which results in the spontaneous formation of large‐scale, continuous nanometer‐thick Ti_3_C_2_T_x_ films at the liquid/liquid interface.

**Figure 2:**
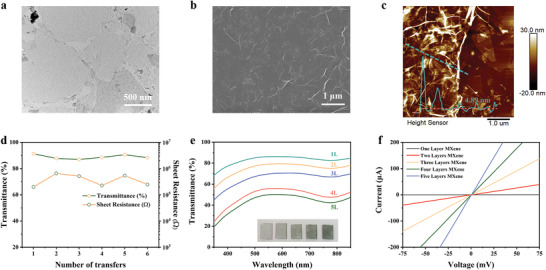
a) Typical TEM image, b) SEM image, and c) AFM image and line‐scan height analysis of the assembled monolayer Ti_3_C_2_T_x_ films, respectively. d) Optical transmittance at 550 nm and sheet resistance of the Ti_3_C_2_T_x_ films transferred onto SiO_2_ substrates from six different batches. e) Optical transmittance spectra (measured with films transferred onto the glass) and f) Current–voltage curve of the as‐prepared multilayered Ti_3_C_2_T_x_ films.

TEM and SEM characterizations were further carried out to obtain more morphological information and homogeneity of the interfacial assembled Ti_3_C_2_T_x_ films. We transferred the as‐prepared Ti_3_C_2_T_x_ films to the copper mesh and anodized aluminum oxide (AAO) for subsequent characterizations, respectively. As shown by TEM image in **Figure** **2**a, large area homogeneous and transparent Ti_3_C_2_T_x_ films with nanometer‐scale thickness are obtained by the controlled interfacial assembly method. The resultant Ti_3_C_2_T_x_ nanoflakes are all horizontally oriented with a slight overlap between adjacent nanoflakes, exhibiting a stacking morphology with a few layers. The SEM image (Figure 2b) of the Ti_3_C_2_T_x_ films on the AAO membrane further illustrates the ultrathin and homogeneous characteristics of the interfacially assembled films, where the pores of the anodized aluminum oxide below the Ti_3_C_2_T_x_ films can still be distinctly observed. In addition, the presence of some wrinkles on the Ti_3_C_2_T_x_ films was also obviously observed due to the rapid assembly and sample transfer of the nanoflakes at the soft liquid/liquid interface. The fractional area coverage of the nanometer‐thick Ti_3_C_2_T_x_ films is calculated to be 99.7% by using ImageJ software to distinguish the area of the exposed AAO substrate from the Ti_3_C_2_T_x_ film. The nanometer‐scale ultrathin feature of assembled Ti_3_C_2_T_x_ films was further demonstrated by the cross‐sectional SEM images (Figure S6a, Supporting Information) of the Ti_3_C_2_T_x_ films on the AAO membrane where the sharp and thin edges of the Ti_3_C_2_T_x_ films can be clearly observed, confirming the few‐layer‐stacked nature of the films. In addition, we conducted atomic force microscopy (AFM) measurement to obtain the thickness and homogeneity information of the Ti_3_C_2_T_x_ films. Figure 2c shows the AFM image of the fringes region of the Ti_3_C_2_T_x_ films, where nanoflakes are planarly stacked in a homogeneous manner creating few‐layer‐stacked rough films with obvious wrinkles on it, which is consistent with the results observed by SEM and TEM. The thicknesses of monolayered and bilayered Ti_3_C_2_T_x_ nanoflakes measured from AFM step scans were ≈2.2 and 4.5 nm respectively (Figure S7a,b, Supporting Information), which agrees well with the results previously reported.^[^ ^27^ ^]^ The average thicknesses of 4.89 nm for the Ti_3_C_2_T_x_ thin films were obtained excluding the obvious wrinkled areas of the films, which roughly corresponds to two or three layers of the Ti_3_C_2_T_x_ nanoflakes.

The interlayer overlaps between Ti_3_C_2_T_x_ nanoflakes are critical to the electrical conductivity and transmittance of the resulting nanofilms. The optical and electronic properties of the Ti_3_C_2_T_x_ films prepared onto the Au gap electrodes patterned on the glass substrate surface were then evaluated by UV–vis spectrophotometry and current–voltage curve measurements. Firstly, we compared the effect of the dispersion concentration of Ti_3_C_2_T_x_ nanoflakes on the transmittance of the assembled films and the results are shown in Figure S8a (Supporting Information). The transmittance of the Ti_3_C_2_T_x_ nanofilms at 550 nm reaches 91.31% when the dispersion concentration is 0.005 mg mL^−1^ and decreases steadily to 70.13% as the dispersion concentration increases to 0.04 mg mL^−1^, as high concentration of Ti_3_C_2_T_x_ nanoflakes causes irregular and multilayered stacking of the resulting films. The reproducibility of the interfacial assembly method was also confirmed by measuring the transmittance and resistance of the prepared Ti_3_C_2_T_x_ film samples from six different batches. As shown in Figure 2d, the transmittance of six different batches of samples at 550 nm is maintained at around 90%, and their resistance values though exhibit significant differences from each other but still remain in the same order of magnitude (10^5^ Ω), demonstrating excellent reproducibility of the method for preparing Ti_3_C_2_T_x_ nanofilms. The thickness of the resulting films can be easily controlled by successive deposition of monolayer films onto a single substrate. The change of color from dark blue to greenish resulting from the variation of multiple layers of Ti_3_C_2_T_x_ films can be visually observed as shown in the inset image of Figure 2e. Figure 2e,f shows the transmission spectra and current–voltage curve of the resulting multilayered Ti_3_C_2_T_x_ films on the SiO_2_ substrate. The corresponding transmittance at 550 nm and resistance values of multilayered Ti_3_C_2_T_x_ films are presented in Figure S8b,c (Supporting Information). After five successive depositions, the optical transmittance (at 550 nm) gradually decreased from 90% to ≈49%. The relationship between the number of depositions and the transmittance showed an approximately linear dependence, indicating that the layer number and thickness of Ti_3_C_2_T_x_ films stacked in each deposition cycle are roughly the same. The average value of the resistances of multiple deposited Ti_3_C_2_T_x_ films decays rapidly from 396 910 to 209 Ω with increasing thickness of the films. Figure S9a (Supporting Information) presents the transmittance at 550 nm versus the resistance of the transparent conductive Ti_3_C_2_T_x_ films, fabricated by previously reported different methods and the interfacial assembly approach. The resistance of the assembled Ti_3_C_2_T_x_ films reaches 1867 Ω at a transmittance of 79%, 540 Ω at 70%, and 279 Ω at 56%. The resistances of the interfacial assembled films at the same transmittance are lower than MXene films made by spray coating (7974 Ω at 82%) and Langmuir–Blodgett technology (2960 Ω at 88%), but still higher than films prepared by spin coating (201 Ω at 87%), and similar to the resistance of films prepared by the other assembly methods (650 Ω at 79%).^[^ ^26^, ^48^, ^49^, ^50^, ^51^, ^52^, ^53^ ^]^ Given the convenience and effectiveness of the interfacial assembly method, the interfacially assembled MXene films show unique advantages compared to other fabrication methods and ha significant potential for optoelectronic applications.

Large‐scale nm‐thick ZnIn_2_S_4_ films were prepared by the same method using liquid‐dispersed exfoliated ZnIn_2_S_4_ nanoflakes as precursors. The homogeneity of the ZnIn_2_S_4_ films was relatively worse than the MXene films fabricated by the chemical exfoliation approach due to the inhomogeneity resulting from the ultrasonic exfoliation approach, as further confirmed by TEM, SEM and AFM observations. As shown in TEM image (Figure S10a, Supporting Information), the ZnIn_2_S_4_ nanofilms with horizontally oriented, edge‐to‐edge flake arrangement morphology are confirmed to be successfully constructed from inhomogeneity mechanically exfoliated ZnIn_2_S_4_ nanoflakes. The SEM image of the large‐area ZnIn_2_S_4_ nanofilms on the AAO substrate (Figure S10b, Supporting Information) further confirms the reliability and extensibility of the interfacial assembly method for the fabrication of membranes of different 2D materials with nanoscale thicknesses. The fractional area coverage value of nm‐thick ZnIn_2_S_4_ films was calculated to be 94.9% by using ImageJ software. The cross‐sectional SEM image (Figure S6b, Supporting Information) of the ZnIn_2_S_4_ films on AAO membrane revealed the nanometer‐thickness feature of the films. The average thicknesses of 8.04 nm for the as‐prepared ZnIn_2_S_4_ films were obtained from AFM measurement as shown in Figure S10c (Supporting Information), which roughly corresponds to the thickness of four layers of ZnIn_2_S_4_ nanoflakes.^[^ ^54^ ^]^ Our interfacial assembly method accomplished the fabrication of densely packed assembled films with fine nanoscale controllability of film thickness, outstanding uniformity, and high surface coverage, which is difficult to realize with other traditional strategies for fabricating thin films of 2D materials.

### 2.3 Optoelectronic Properties of Ti_3_C_2_T_x_/ZIS Heterostructure

We then fabricated Ti_3_C_2_T_x_/ZIS heterojunction photodetectors for UV–vis detection by successive deposition of interfacial assembled Ti_3_C_2_T_x_ nanofilms and ZnIn_2_S_4_ nanofilms onto the SiO_2_ substrate. The gold pad electrodes fabricated by the “lift‐off, float‐on” (LOFO) technique were used as soft contacts for *I*–*V* measurements. The details can be found in the Experimental Section (Supporting Information). The optical image and schematic diagram of the as‐fabricated Ti_3_C_2_T_x_/ZnIn_2_S_4_ heterojunction photodetector were illustrated in Figure S11a,b (Supporting Information), where ZnIn_2_S_4_ and Ti_3_C_2_T_x_ films were deposited on two separate sides of the SiO_2_ substrate with an overlap width of 1 mm in between. Firstly, we investigated the relationship between the number of deposition of the corresponding films and the photodetection performance of the resulting Ti_3_C_2_T_x_/ZIS heterojunction photodetector. The deposition number of the Ti_3_C_2_T_x_ films was determined to be 5 layers for the following experiments as the change of the resistance of Ti_3_C_2_T_x_ films was relatively little when exceeding 5 layers. As for ZnIn_2_S_4_, as shown in Figure S12a (Supporting Information), the photodetection performance of the device increases with increasing the layer number of ZnIn_2_S_4_ films up to 7 layers then starts to decrease due to progressively more interlayer defects inducing recombination of the photogenerated free carriers. The photodetector has an ultralow dark current in the magnitude of 10^–12^ A which does not significantly change with the layer number of ZnIn_2_S_4_ films due to relatively low dark current characteristic of the ZnIn_2_S_4_. The observed highest photocurrent response of the as‐prepared Ti_3_C_2_T_x_/ZIS heterojunction photodetector was obtained from 7‐layer ZnIn_2_S_4_ films under 450 nm laser illumination at a power density of 190 mW cm^−2^.

**Figure 3:**
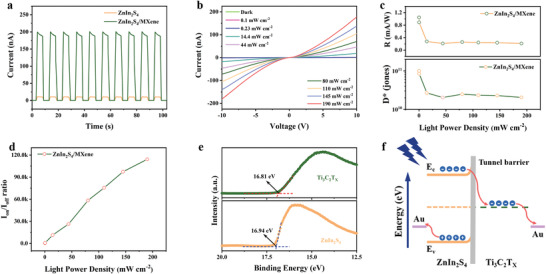
a) Current–time curves of the Ti_3_C_2_T_x_/ZIS and the pristine ZIS device with the deposition of 7‐layer ZnIn_2_S_4_ films recorded at 10 V bias under a 450 nm light illumination. b) Linear *I*–*V* characteristics of the Ti_3_C_2_T_x_/ZIS heterojunction photodetector under a 450 nm light illumination with different light intensities. c) Specific detectivity and responsivity were measured at 10 V bias under a 450 nm light illumination with different light intensities. d) Ratios of the photocurrent to the dark current of Ti_3_C_2_T_x_/ZIS heterojunction photodetector recorded at 10 V bias under a 450 nm light illumination with different light intensities. e) The magnified region from 20 to 12.5 eV of UPS spectra to determine the secondary electron cut‐off energy of Ti_3_C_2_T_x_ and ZnIn_2_S_4_ nanoflakes. f) Energy band diagram of the Ti_3_C_2_T_x_/ZIS heterojunction.

Next, the voltage‐dependent photoresponse of the heterojunction devices was investigated. As shown in Figure S12b (Supporting Information), the Ti_3_C_2_T_x_/ZIS photodetectors generated definite photoswitching characteristics at a wide range of bias voltages. Specifically, the positive correlation between photocurrent and bias voltage provides a flexible modulation of the optical response for versatile applications. **Figure** **3**a shows typical photoresponse curves of the Ti_3_C_2_T_x_/ZIS heterojunction photodetector and the pristine ZIS photodetector with the deposition of 7‐layer ZnIn_2_S_4_ films. The photocurrent of the Ti_3_C_2_T_x_/ZIS photodetector can reach ≈200 nA, which is ≈20 times higher than that of the pristine ZIS photodetector upon the same illumination condition at a bias of 10 V. The enhancement of photocurrent for the heterojunction photodetectors can be primarily attributed to the efficient separation and transport of photogenerated carriers with the benefit of the excellent electrical conductivity of Ti_3_C_2_T_x_ nanoflakes. The specific detectivity and responsivity of the as‐fabricated Ti_3_C_2_T_X_/ZIS heterojunction photodetector under illumination of different wavelengths were further examined. As shown in Figure S12c (Supporting Information), the specific detectivity and responsivity of the device decrease with increasing laser wavelength under the 10 V bias, indicating that the photodetector has a superior response to laser irradiation with shorter wavelengths, which is also well consistent with the UV–vis diffuse reflectance spectra (UV–vis DRS) of the liquid‐dispersed ZnIn_2_S_4_ nanoflakes (Figure S14a, Supporting Information). Figure 3b exhibits the current–voltage curves of the photodetector under 450 nm laser illumination with the light intensity varying from dark to 190 mW cm^−2^ recorded from −10 to +10 V. The photocurrent increases from 1.7 pA to 182 nA as the light intensity increases from dark to 190 mW cm^−2^, demonstrating high detection sensitivity, with the high light on/off ratio around 10^5^ of the device. It is worth noting that the *I*–*V* curve of the Ti_3_C_2_T_x_/ZIS heterojunction photodetector displayed linearly symmetric Ohmic contact characteristics which are significantly different from the Schottky contact between ZnIn_2_S_4_ and Ti_3_C_2_T_x_ previously reported.^[^ ^40^ ^]^

To further evaluate the optoelectronic performance of the as‐fabricated Ti_3_C_2_T_x_/ZIS heterojunction photodetector, some critical parameters such as responsivity (*R*), specific detectivity (D*), and on/off ratio (*I*_ph_/*I*_dark_) were calculated via formulas given below:

(1) $$R=\frac{I_{\mathrm{light}}-I_{\mathrm{dark}}}{P_{\mathrm{light}}A}\left[A\cdot W^{-1}\right]$$

(2) $$D^{*}=\frac{R\sqrt{A}}{\sqrt{2eI_{\mathrm{dark}}}}\left[\mathrm{cm}\cdot {\mathrm{Hz}}^{1/2}\cdot W^{-1}\left(\mathrm{Jones}\right)\right]$$

where *I*_light_ and *I*_dark_ represent the current under light and dark conditions, respectively; *P*_light_ is the incident light power density; *A* is the effective area of the device (≈0.5 mm^2^, details are provided in the Device Characterization of Experimental Section, Supporting Information); *e* is the elementary charge.^[^ ^30^ ^]^ Figure 3c presents the responsivity and detectivity of the photodetectors under various incident light power densities at a bias of 10 V. There is a decreasing tendency of *R* and D* with increasing incident light power density owing to the increasing possibility of hole–electron pair recombination with high‐intensity irradiation. The maximum responsivity and detectivity of the Ti_3_C_2_T_x_/ZIS heterojunction photodetector were calculated up to 1 mA W^–1^ and 1.04 × 10^11^ Jones, respectively, under the illumination of 450 nm light with a power density at 0.1 mW cm^−2^. The corresponding relationship between the on/off ratio with various incident light power densities is shown in Figure 3d, from which the on/off ratio of the device increases almost linearly with increasing laser power densities owing to the increasing photogenerated electron−hole pairs. Benefiting from the ultra‐low dark current of the ZnIn_2_S_4_ films, the as‐fabricated heterojunction photodetector exhibits a very significant light on/off ratio of around 10^5^ at a bias of 10 V, indicating that the photodetector has an excellent resolution to distinguish the optical signals from the noise environment. The rise and decay time (*τ*_rise_ and *τ*_decay_, defined as the time intervals between 10% and 90% of the peak photocurrent) can be measured to be *τ*_rise_ = 646.8 ms and *τ*_decay_ = 640 ms from the single photoswitching response cycle as shown in Figure S12d (Supporting Information). Based on the above results, the fabricated Ti_3_C_2_T_x_/ZIS heterojunction photodetector fabricated by the interfacial assembly strategy has excellent optoelectronic characteristics, which is superior to most of the previously reported photodetectors made of 2D materials based on liquid‐phase‐exfoliated strategy, and are even comparable to some previous work of photodetectors fabricated by chemical vapor deposition (CVD), pulsed laser deposition (PLD), etc. (shown in **Table** **1**). The performance of fabricated Ti_3_C_2_T_x_/ZIS heterojunction might be further promoted using annealing. Then, the stability of the Ti_3_C_2_T_x_/ZIS heterojunction devices in the ambient environment was evaluated. As seen from the photoswitching response curve of the Ti_3_C_2_T_x_/ZIS heterojunction that was stored in air for 7 days as shown in Figure S13 (Supporting Information), the photodetector without encapsulation displayed a relatively stable photoresponse, with maintained ≈95.6% signal after seven days of shelf storage, demonstrating outstanding stability of the device for long‐term applications.

**Table 1:** Performance comparison of the Ti_3_C_2_T_x_/ZIS photodetectors with other photodetectors fabricated by liquid‐phase‐exfoliation strategy and other complex methods (N/A: Not applicable)

Materials	*V*_ds_	*R* [A W^–1^]	D* [Jones]	On/Off ratio	Fabrication methods	Ref.
Ti_3_C_2_T_x_/ZIS	10 V	1.04 × 10^−3^	1 × 10^11^	≈10^5^	Interfacial assembly	This work
MoS_2_/MXene	1 V	1.9	1 × 10^10^	N/A	Solution method	[29]
Zn_2_GeO_4_/MXene	6 V	2 × 10^−2^	N/A	N/A	CVD	[55]
ZnIn_2_S_4_	2 V	1.4	9.8 × 10^9^	>1000	PLD	[38]
MoS_2_	10 V	16	4 × 10^12^	900	Liquid phase exfoliation	[56]
MoS_2_	10 V	5 × 10^−2^	3.18 × 10^9^	N/A	Inkjet‐printed	[57]
MoS_2_	5 V	0.57	≈10^10^	N/A	CVD	[58]
MoS_2_/Glassy‐graphene	1 V	12.3 × 10^−3^	1.8 × 10^10^	N/A	Polymer‐assisted deposition (PAD)	[59]
MoS_2_/g‐C_3_N_4_	10 V	0.7	8 × 10^10^	10^4^	Liquid phase exfoliation	[60]
WSe_2_/Bi_2_O_2_Se	5 V	0.284	N/A	10^5^	CVD	[61]
WSe_2_/*α*‐In_2_Se_3_	1 V	1.84	1.34 × 10^11^	N/A	CVD	[62]

To further investigate the mechanism of the separation and transport of photogenerated carriers at the heterojunction interface between MXene and ZnIn_2_S_4_ nanofilms, ultraviolet photoelectron spectroscopy (UPS), Ultraviolet–visible diffuse reflectance spectroscopy (UV–vis DRS), and X‐ray photoelectron valence band spectra (XPS‐VB) were implemented to obtain the energy band alignment of the Ti_3_C_2_T_x_/ZIS heterojunction. The survey UPS spectrum and corresponding secondary electron cut‐off edge are shown in Figure S14c,d (Supporting Information) and Figure 3e. The work functions of ZnIn_2_S_4_ and Ti_3_C_2_T_x_ were determined to be 4.28 and 4.41 eV, respectively, by subtracting the secondary electron cut‐off energy from the incident ultraviolet photon energy of the He I light source (21.22 eV), which is consistent with previously reported values.^[^ ^31^, ^63^ ^]^ The difference between the VB maximum and Fermi energy of ZnIn_2_S_4_ was estimated to be 1.24 eV, which is determined from the intersection of the linear extrapolation and the baseline of the XPS‐VB spectra as shown in Figure S15a (Supporting Information), indicating the *n*‐type feature of the ZnIn_2_S_4_. The UV–vis absorption diffuse reflectance spectra of ZnIn_2_S_4_ are presented in Figure S14a (Supporting Information), which demonstrates the strong and wide visible‐light‐harvesting capability of the ZnIn_2_S_4_ in the visible range from 300 to 550 nm. The corresponding energy band gap of ZnIn_2_S_4_ was obtained to be 2.34 eV through the Tauc plot in Figure S14b (Supporting Information). In addition, as presented in Figure S16 (Supporting Information), the work function of Au pad electrodes is calculated as 5.1 eV, which was consistent with the previously reported values.^[^ ^64^ ^]^ Based on the above results, the band alignment of the Ti_3_C_2_T_x_/ZIS heterojunction is shown in Figure 3f. The work function of Ti_3_C_2_T_x_ film is slightly higher than that of ZnIn_2_S_4_, which is expected to form a Schottky junction. However, the current–voltage curve of the Ti_3_C_2_T_x_/ZIS heterojunction photodetector exhibits no significant rectification behaviors. It may be attributed to the weak vdW interaction at the heterojunction interface between MXene and ZnIn_2_S_4_ films, which can significantly alleviate the Fermi level pinning (FLP) effect and result in the generation of additional tunneling barriers at the heterojunction interface to reduce the Schottky barrier.^[^ ^65^, ^66^, ^67^ ^]^ The tunnel potential barrier becomes a major factor affecting the electrical properties of the heterojunction. This situation is similar to the contact between the metals and the highly electrostatically doped semiconductors, which facilitates carrier injection through the interlayer electron tunneling process and exhibits linearly symmetric Ohmic contact characteristics. Photogenerated carriers in Ti_3_C_2_T_x_/ZIS heterojunction are separated, driven by an applied voltage and collected by Au electrodes. In addition, benefiting from the excellent electrical conductivity of Ti_3_C_2_T_x_ nanoflakes and superior light‐harvesting capability of ZnIn_2_S_4_, the photogenerated carriers at the interface of Ti_3_C_2_T_x_/ZIS heterojunction can be generated and transported more efficiently, leading to the significant increase of the photodetection performance of the photodetectors.

## 3 Conclusions

In summary, we fabricated a high‐performance Ti_3_C_2_T_x_/ZIS heterojunction photodetector through the interfacial assembly of the exfoliated MXene and ZnIn_2_S_4_ nanoflakes at the water/*n*‐hexane interface. The morphology information of corresponding few‐layered assembled films has been characterized by transmission electron microscopy (TEM), scanning electron microscopy (SEM), and atomic force microscopy (AFM). The synergistic interaction between MXene and ZnIn_2_S_4_ heterostructure facilitates the generation and transport of photogenerated carriers more efficiently. The as‐prepared Ti_3_C_2_T_x_/ZIS device has excellent optoelectronic performance including good responsivity (1.04 mA W^−1^), superior detectivity (1 × 10^11^ Jones), huge on/off ratio (≈10^5^), and specific response time (646.8 ms) and recovery time (640 ms), surpasses most reported photodetectors made of 2D materials and fabricated by other methods, demonstrating promising application potential of the devices for low‐cost, reliable, and high‐performance photodetectors. Besides, our interfacial assembly strategy also provides a feasible and prospective way to produce high quality few‐layered 2D materials films for diverse applications without the need for specialized instruments.

## Conflict of Interest

The authors declare no conflict of interest.

## Supporting information

Supporting information

## Data Availability

The data that support the findings of this study are available from the corresponding author upon reasonable request.

## References

[1] B. Nabet , Photodetectors: Materials, Devices, and Applications, Woodhead, UK 2015.

[2] S. Kim , Y. T. Lim , E. G. Soltesz , A. M. De Grand , J. Lee , A. Nakayama , J. A. Parker , T. Mihaljevic , R. G. Laurence , D. M. Dor , L. H. Cohn , M. G. Bawendi , J. V. Frangioni , Nat. Biotechnol. 2004, 22, 93. 14661026 10.1038/nbt920 PMC2346610

[3] T. Yan , Z. Li , F. Cao , J. Chen , L. Wu , X. Fang , Adv. Mater. 2022, 34, 2201303. 10.1002/adma.202201303 35653221

[4] S. J. Liang , B. Cheng , X. Cui , F. Miao , Adv. Mater. 2020, 32, 1903800. 10.1002/adma.201903800 31608514

[5] X. Yu , X. Wang , F. Zhou , J. Qu , J. Song , Adv. Funct. Mater. 2021, 31, 2104260.

[6] S. Liu , L. Zheng , P. Yu , S. Han , X. Fang , Adv. Funct. Mater. 2016, 26, 3331.

[7] Y. Z. Chen , S. W. Wang , T. Y. Su , S. H. Lee , C. W. Chen , C. H. Yang , K. Wang , H. C. Kuo , Y. L. Chueh , Small 2018, 14, 1704052. 10.1002/smll.201704052 29707890

[8] Y. Z. Chen , Y. T. You , P. J. Chen , D. Li , T. Y. Su , L. Lee , Y. C. Shih , C. W. Chen , C. C. Chang , Y. C. Wang , C. Y. Hong , T. C. Wei , J. C. Ho , K. H. Wei , C. H. Shen , Y. L. Chueh , ACS Appl. Mater. Interfaces 2018, 10, 35477. 30107132 10.1021/acsami.8b11676

[9] W. Yang , J. Chen , Y. Zhang , Y. Zhang , J. H. He , X. Fang , Adv. Funct. Mater. 2019, 29, 1808182.

[10] X. Guan , X. Yu , D. Periyanagounder , M. R. Benzigar , J. K. Huang , C. H. Lin , J. Kim , S. Singh , L. Hu , G. Liu , D. Li , J. H. He , F. Yan , Q. J. Wang , T. Wu , Adv. Opt. Mater. 2020, 9, 2001708.

[11] Z. Liu , Y. Zhu , J. K. El‐Demellawi , D. B. Velusamy , A. M. El‐Zohry , O. M. Bakr , O. F. Mohammed , H. N. Alshareef , ACS Energy Lett. 2019, 4, 2315.

[12] H. P. Wang , S. Li , X. Liu , Z. Shi , X. Fang , J. H. He , Adv. Mater. 2021, 33, 2003309. 10.1002/adma.202003309 33346383

[13] A. M. Alamri , S. Leung , M. Vaseem , A. Shamim , J. H. He , IEEE Trans. Electron Devices 2019, 66, 2657.

[14] G. Hu , J. Kang , L. W. T. Ng , X. Zhu , R. C. T. Howe , C. G. Jones , M. C. Hersam , T. Hasan , Chem. Soc. Rev. 2018, 47, 3265. 29667676 10.1039/c8cs00084k

[15] A. G. Kelly , D. O'Suilleabhain , C. Gabbett , J. N. Coleman , Nat. Rev. Mater. 2021, 7, 217.

[16] F. I. Alzakia , S. C. Tan , Adv. Sci. 2021, 8, 2003864. 10.1002/advs.202003864 PMC8188210 34105282

[17] H. Kaur , J. N. Coleman , Adv. Mater. 2022, 2202164. 10.1002/adma.202202164 35470487

[18] X. Cai , Y. Luo , B. Liu , H. M. Cheng , Chem. Soc. Rev. 2018, 47, 6224. 29905344 10.1039/c8cs00254a

[19] C. Zhang , J. Energy Chem. 2021, 60, 417.

[20] Z. Xie , B. Zhang , Y. Ge , Y. Zhu , G. Nie , Y. Song , C. K. Lim , H. Zhang , P. N. Prasad , Chem. Rev. 2022, 122, 1127. 34780169 10.1021/acs.chemrev.1c00165

[21] A. VahidMohammadi , J. Rosen , Y. Gogotsi , Science 2021, 372, 1165. 10.1126/science.abf1581 34112665

[22] D. Zhang , D. Shah , A. Boltasseva , Y. Gogotsi , ACS Photonics 2022, 9, 1108.

[23] H. Xu , A. Ren , J. Wu , Z. Wang , Adv. Funct. Mater. 2020, 30, 2000907.

[24] R. Li , X. Ma , J. Li , J. Cao , H. Gao , T. Li , X. Zhang , L. Wang , Q. Zhang , G. Wang , C. Hou , Y. Li , T. Palacios , Y. Lin , H. Wang , X. Ling , Nat. Commun. 2021, 12, 1587. 33707439 10.1038/s41467-021-21852-7 PMC7952574

[25] M. Mojtabavi , A. VahidMohammadi , W. Liang , M. Beidaghi , M. Wanunu , ACS Nano 2019, 13, 3042. 30844249 10.1021/acsnano.8b08017

[26] S. J. Kim , J. Choi , K. Maleski , K. Hantanasirisakul , H. T. Jung , Y. Gogotsi , C. W. Ahn , ACS Appl. Mater. Interfaces 2019, 11, 32320. 31405272 10.1021/acsami.9b12539

[27] M. Mojtabavi , A. VahidMohammadi , K. Ganeshan , D. Hejazi , S. Shahbazmohamadi , S. Kar , A. C. T. van Duin , M. Wanunu , ACS Nano 2021, 15, 625. 33405898 10.1021/acsnano.0c06393

[28] D. Xiong , W. Deng , G. Tian , B. Zhang , S. Zhong , Y. Xie , T. Yang , H. Zhao , W. Yang , Nano Energy 2022, 93, 106889.

[29] J. Zhu , H. Wang , L. Ma , G. Zou , Nano Res. 2021, 14, 3416.

[30] W. Song , Q. Liu , J. Chen , Z. Chen , X. He , Q. Zeng , S. Li , L. He , Z. Chen , X. Fang , Small 2021, 17, 2100439. 10.1002/smll.202100439 33891802

[31] Z. Kang , Y. Ma , X. Tan , M. Zhu , Z. Zheng , N. Liu , L. Li , Z. Zou , X. Jiang , T. Zhai , Y. Gao , Adv. Electron. Mater. 2017, 3, 1700165.

[32] C. Yi , Y. Chen , Z. Kang , Y. Ma , Y. Yue , W. Liu , M. Zhu , Y. Gao , Adv. Electron. Mater. 2021, 7, 2000955.

[33] W. Song , J. Chen , Z. Li , X. Fang , Adv. Mater. 2021, 33, 2101059. 10.1002/adma.202101059 34046946

[34] W. Ouyang , J. Chen , J. H. He , X. Fang , Adv. Electron. Mater. 2020, 6, 2000168.

[35] J. Chen , X. Liu , Z. Li , F. Cao , X. Lu , X. Fang , Adv. Funct. Mater. 2022, 32, 2201066.

[36] L. Valdman , V. Mazánek , P. Marvan , M. Serra , R. Arenal , Z. Sofer , Adv. Opt. Mater. 2021, 9, 2100845.

[37] W. Zhen , X. Zhou , S. Weng , W. Zhu , C. Zhang , ACS Appl. Mater. Interfaces 2022, 14, 12571. 35234462 10.1021/acsami.2c00063

[38] Q. Ye , J. Lu , Z. Zheng , W. Huang , J. Yao , G. Yang , Adv. Opt. Mater. 2022, 10, 2102335.

[39] T. Ren , H. Huang , N. Li , D. Chen , Q. Xu , H. Li , J. He , J. Lu , J. Colloid Interface Sci. 2021, 598, 398. 33930744 10.1016/j.jcis.2021.04.027

[40] G. Zuo , Y. Wang , W. L. Teo , A. Xie , Y. Guo , Y. Dai , W. Zhou , D. Jana , Q. Xian , W. Dong , Y. Zhao , Angew. Chem., Int. Ed. 2020, 59, 11287. 10.1002/anie.202002136 32250502

[41] M. S. Javed , X. Zhang , S. Ali , A. Mateen , M. Idrees , M. Sajjad , S. Batool , A. Ahmad , M. Imran , T. Najam , W. Han , Nano Energy 2022, 101, 107624.

[42] C. F. J. Zhang , S. Pinilla , N. McEyoy , C. P. Cullen , B. Anasori , E. Long , S. H. Park , A. Seral‐Ascaso , A. Shmeliov , D. Krishnan , C. Morant , X. H. Liu , G. S. Duesberg , Y. Gogotsi , V. Nicolosi , Chem. Mater. 2017, 29, 4848.

[43] Z. Li , X. Liu , C. Zuo , W. Yang , X. Fang , Adv. Mater. 2021, 33, 2103010. 10.1002/adma.202103010 34431141

[44] K. Hantanasirisakul , M. Alhabeb , A. Lipatov , K. Maleski , B. Anasori , P. Salles , C. Ieosakulrat , P. Pakawatpanurut , A. Sinitskii , S. J. May , Y. Gogotsi , Chem. Mater. 2019, 31, 2941.

[45] Y. Cao , Q. Deng , Z. Liu , D. Shen , T. Wang , Q. Huang , S. Du , N. Jiang , C.‐T. Lin , J. Yu , RSC Adv. 2017, 7, 20494.

[46] B. Lyu , M. Kim , H. Jing , J. Kang , C. Qian , S. Lee , J. H. Cho , ACS Nano 2019, 13, 11392. 31553884 10.1021/acsnano.9b04731

[47] T. Zhang , T. Wang , F. Meng , M. Yang , S. Kawi , J. Mater. Chem. 2022, 10, 5400.

[48] J. Wu , H. A. Becerril , Z. Bao , Z. Liu , Y. Chen , P. Peumans , Appl. Phys. Lett. 2008, 92, 263302.

[49] K. Hantanasirisakul , M. Q. Zhao , P. Urbankowski , J. Halim , B. Anasori , S. Kota , C. E. Ren , M. W. Barsoum , Y. Gogotsi , Adv. Electron. Mater. 2016, 2, 1600050.

[50] G. M. Weng , J. Li , M. Alhabeb , C. Karpovich , H. Wang , J. Lipton , K. Maleski , J. Kong , E. Shaulsky , M. Elimelech , Y. Gogotsi , A. D. Taylor , Adv. Funct. Mater. 2018, 28, 1803360.

[51] C. J. Zhang , B. Anasori , A. Seral‐Ascaso , S. H. Park , N. McEvoy , A. Shmeliov , G. S. Duesberg , J. N. Coleman , Y. Gogotsi , V. Nicolosi , Adv. Mater. 2017, 29, 1702678. 10.1002/adma.201702678 28741695

[52] G. Ying , A. D. Dillon , A. T. Fafarman , M. W. Barsoum , Mater. Res. Lett. 2017, 5, 391.

[53] H. A. Becerril , J. Mao , Z. Liu , R. M. Stoltenberg , Z. Bao , Y. Chen , ACS Nano 2008, 2, 463. 19206571 10.1021/nn700375n

[54] M. Q. Yang , Y. J. Xu , W. Lu , K. Zeng , H. Zhu , Q. H. Xu , G. W. Ho , Nat. Commun. 2017, 8, 14224. 28146147 10.1038/ncomms14224 PMC5296640

[55] S. Guo , S. Kang , S. Feng , W. Lu , J. Phys. Chem. 2020, 124, 4764.

[56] D. B. Velusamy , R. H. Kim , S. Cha , J. Huh , R. Khazaeinezhad , S. H. Kassani , G. Song , S. M. Cho , S. H. Cho , I. Hwang , J. Lee , K. Oh , H. Choi , C. Park , Nat. Commun. 2015, 6, 8063. 26333531 10.1038/ncomms9063 PMC4569699

[57] J.‐W. T. Seo , J. Zhu , V. K. Sangwan , E. B. Secor , S. G. Wallace , M. C. Hersam , ACS Appl. Mater. Interfaces 2019, 11, 5675. 30693759 10.1021/acsami.8b19817

[58] D.‐S. Tsai , K.‐K. Liu , D.‐H. Lien , M.‐L. Tsai , C.‐F. Kang , C.‐A. Lin , L.‐J. Li , J.‐H. He , ACS Nano 2013, 7, 3905. 23590667 10.1021/nn305301b

[59] H. Xu , X. Han , X. Dai , W. Liu , J. Wu , J. Zhu , D. Kim , G. Zou , K. A. Sablon , A. Sergeev , Z. Guo , H. Liu , Adv. Mater. 2018, 30, 1706561. 10.1002/adma.201706561 29380432

[60] D. B. Velusamy , M. A. Haque , M. R. Parida , F. Zhang , T. Wu , O. F. Mohammed , H. N. Alshareef , Adv. Funct. Mater. 2017, 27, 1605554.

[61] P. Luo , F. Wang , J. Qu , K. Liu , X. Hu , K. Liu , T. Zhai , Adv. Funct. Mater. 2021, 31, 2008351.

[62] J. Zou , Y. Ke , X. Zhou , Y. Huang , W. Du , L. Lin , S. Wei , L. Luo , H. Liu , C. Li , K. Shen , A. Ren , J. Wu , Adv. Opt. Mater. 2022, 10, 2200143.

[63] C. Du , B. Yan , Z. Lin , G. Yang , J. Mater. Chem. 2020, 8, 207.

[64] C. Xu , C. Li , Y. Jin , Adv. Electron. Mater. 2022, 8, 2101251.

[65] Y. Zheng , J. Gao , C. Han , W. Chen , Cell Rep. Phys. Sci. 2021, 2, 100298.

[66] T. Shen , J. C. Ren , X. Liu , S. Li , W. Liu , J. Am. Chem. Soc. 2019, 141, 3110. 30688068 10.1021/jacs.8b12212

[67] A. Allain , J. Kang , K. Banerjee , A. Kis , Nat. Mater. 2015, 14, 1195. 26585088 10.1038/nmat4452

