# Supplementary material for: Interfacial Assembly of Ti3C2Tx/ZnIn2S4 Heterojunction for High‐Performance Photodetectors
Source: Adv Sci (Weinh). 2022 Oct 26;9(35):2204687. doi: 10.1002/advs.202204687 (PMC9762283; doi:10.1002/advs.202204687)
Supplement: Supplementary file 1 — Supporting information [file ADVS-9-2204687-s001.pdf]

ADVANCED SCIENCE
Open Access

Supporting Information

for *Adv. Sci.*, DOI 10.1002/advs.202204687

Interfacial Assembly of $Ti_3C_2T_x/ZnIn_2S_4$ Heterojunction for High-Performance Photodetectors

*Shuping Hou, Chen Xu, Xingkai Ju and Yongdong Jin**

Supporting Information

Interfacial Assembly of $Ti_3C_2T_x/ZnIn_2S_4$ Heterojunction for High-Performance Photodetectors

*Shuping Hou, Chen Xu, Xingkai Ju, and Yongdong Jin**

State Key Laboratory of Electroanalytical Chemistry, Changchun Institute of Applied Chemistry, Chinese Academy of Sciences, Changchun 130022, China; School of Applied Chemistry and Engineering, University of Science and Technology of China, Hefei 230026 Anhui, China

E-mail: ydjin@ciac.ac.cn

Experimental section

Materials: Ti_3AlC_2 (200 mesh, 98%), zinc chloride ($ZnCl_2$, 99%) and thioacetamide (TAA, 99%) were obtained from Aladdin, lithium fluoride (LiF, 99.9%) and indium chloride tetrahydrate ($InCl_3·4H_2O$, 99%) were purchased from Macklin. Hydrochloric acid, methanol, and n-hexane were obtained from Xilong Scientific Co. Ltd. Water used throughout all these experiments was purified with a Millipore system (18.2 MΩ·cm).

Synthesis of Exfoliated $Ti_3C_2T_x$ Nanoflakes: LiF (1 g) was dissolved in 10 mL of HCl (9 $mol·L^{-1}$) completely by continuous stirring in the Teflon tube (50 mL) at 35 °C in a water heating bath, and then 0.5 g of Ti_3AlC_2 was added with gentle stirring. The reaction was allowed to continue for 24 h with 300 rpm stirring at 35 °C. The resulting product was centrifuged (at 3500 rpm for 5 min each cycle) several times with deionized (DI) water until the pH of ~6 is achieved. Finally, the dark green $Ti_3C_2T_x$ MXene aqueous solution was obtained from the supernatant after centrifugation at 3500 rpm for an hour for further use.

Synthesis of Exfoliated $ZnIn_2S_4$ Nanoflakes: $ZnIn_2S_4$ nanoflowers were fabricated by a facile low-temperature refluxing method followed by a moderate exfoliation. In detail, 0.3 mmol of $ZnCl_2$ and 0.6 mmol of $InCl_3·4H_2O$ were added to 30 ml DI water and stirred for 30 min. Subsequently, an excess amount of thioacetamide (TAA, 1.8 mmol) was added to the above solution and stirred for another 30 min. The solution was then heated to 95 °C and maintained at that temperature for 5 h under vigorous stirring. The resulting precipitation was collected by centrifugation 2 times and re-dispersed into 50 ml DI water. The dispersion was sonicated continuously for 2 hours and then centrifuged at 5000 rpm for 5min to remove aggregates. After that, $ZnIn_2S_4$ nanoflakes were obtained from the supernatant after centrifugation.

Fabrication of Assembled $Ti_3C_2T_x$ ($ZnIn_2S_4$) Films: The assembled MXene thin Films were prepared by the method of liquid/liquid interface assembly. Glass substrate was ultrasonic cleaned in acetone, ethanol, and water sequentially for the next operation. Firstly, 3 mL of the prepared $Ti_3C_2T_x$ nanoflakes solution (0.002 mg/mL), 0.5 mL of n-hexane, and 200 μL of hydroxyl acid (1M) were mixed to form the liquid-liquid interface and reduce electrostatic repulsion between individual 2D $Ti_3C_2T_x$ nanoflakes. And then, methanol was injected rapidly into the two-phase interface to induce the dispersed 2D $Ti_3C_2T_x$ nanoflakes being captured at the interface. After evaporation of n-hexane, the nanomembrane was exposed to the "soft" air−water interface and transferred onto the pre-cleaned glass substrate. $ZnIn_2S_4$ Films were fabricated by the same interfacial assembly procedure.

Device Fabrication: By repeating the above process, the multi-layered $Ti_3C_2T_x$/ZIS heterojunction photodetector was constructed. After drying in air thoroughly, we put Au pads (prepared by thermally evaporating Au on the glass substrate with a shadow mask technique in the vacuum) onto the film to form the $Ti_3C_2T_x$/ZIS heterojunction photodetector, where $ZnIn_2S_4$ and $Ti_3C_2T_x$ were deposited at two separate sides of the substrate with an overlap width of 1 mm in between.

Device characterization: Before device characterization, we scratch the peripheral heterojunction film around a pair of Au electrodes with a sharp probe under microscopic view to standardize the effective area of heterojunction under laser illumination. The effective area of heterojunction devices is the channel area (~ 0.5 mm^2) between Au electrodes, which has a length of 1 mm and a width of 0.5 mm. Device characterization was performed at room temperature under atmospheric conditions by using a probe station equipped with a Keithley 2636B source meter. W probes connected with Au pad electrodes on the heterojunction film lightly on the probe station as source and drain, respectively. The Xe lamp with different

monochromators and lasers with different wavelengths (650 nm, 532nm and 450 nm) were used to provide continuous illumination.

Materials Characterization: JEM-2100F transmission electron microscope (TEM) and an XL30 ESEM scanning electron microscope (SEM) were used to characterize the morphologies of 2D material nanoflakes and films. SEM samples were prepared on the substrate of the AAO membrane to identify their structures. UV-2600 spectrophotometer (Shimadzu, Japan) was used to obtain the UV-Vis absorption and transmittance spectra of MXene nanoflakes and films. XRD analysis was performed using a Bruker D8 Advanced diffractometer with filtered Cu Kα radiation ($\lambda = 0.154$ nm). AFM imaging was performed using a Dimension Icon (Bruker). X-ray photoelectron spectroscopy (XPS) measurements were carried out on Escalab 250Xi, Thermo Fisher Scientific. Ultraviolet-visible diffuse reflectance spectroscopy (UV–vis DRS) was performed using UV-3600i Plus spectrophotometer (Shimadzu, Japan). Keithley 2636B source meter and VIW-M-1010 probe station from Beijing huazhuo Technology Co. Ltd. were used to record the electrical characteristics of the devices in the ambient condition, and continuous lasers with different wavelengths were used for illumination in this study.

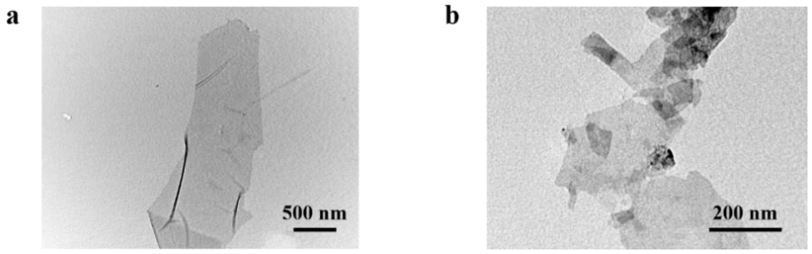

Figure S1. (a, b) Typical TEM images of the liquid exfoliated $Ti_3C_2T_x$ and $ZnIn_2S_4$ nanoflakes.

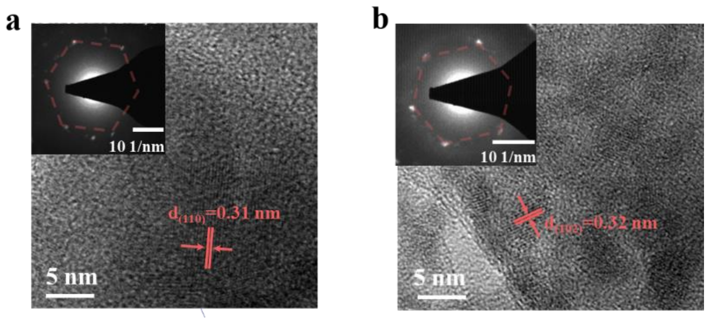

Figure S2. (a, b) The HRTEM images of $Ti_3C_2T_x$ and $ZnIn_2S_4$ nanoflakes, respectively, showing the corresponding SAED patterns of $Ti_3C_2T_x$ and $ZnIn_2S_4$ nanoflakes.

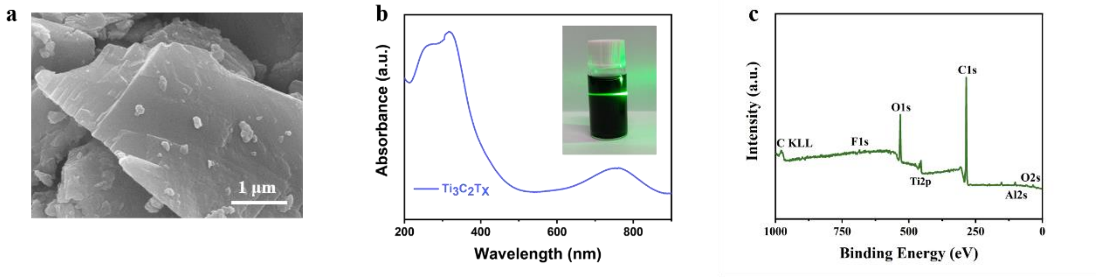

Figure S3. (a) SEM images of unexfoliated Ti_3AlC_2. (b) UV−vis absorbance spectrum of the liquid exfoliated $Ti_3C_2T_x$ nanoflakes dispersion. (c) The survey XPS spectrum of the $Ti_3C_2T_x$ nanoflakes.

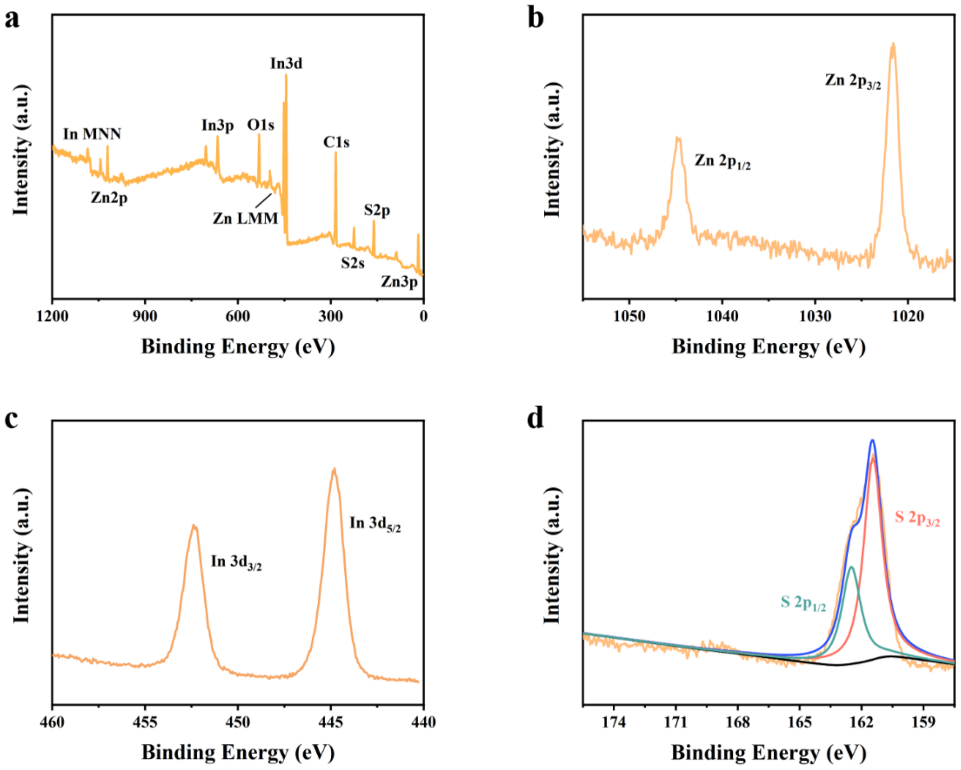

Figure S4. The XPS spectra of (a) survey, and (b-d) high-resolution spectra of Zn 2p, In 3d, and S 2p regions of the exfoliated $ZnIn_2S_4$ nanoflakes, respectively.

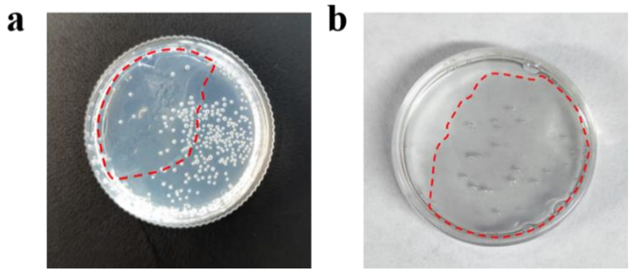

Figure S5. (a, b) The optical image of monolayered $ZnIn_2S_4$ film and $Ti_3C_2T_x$ film at the liquid/liquid interface, respectively.

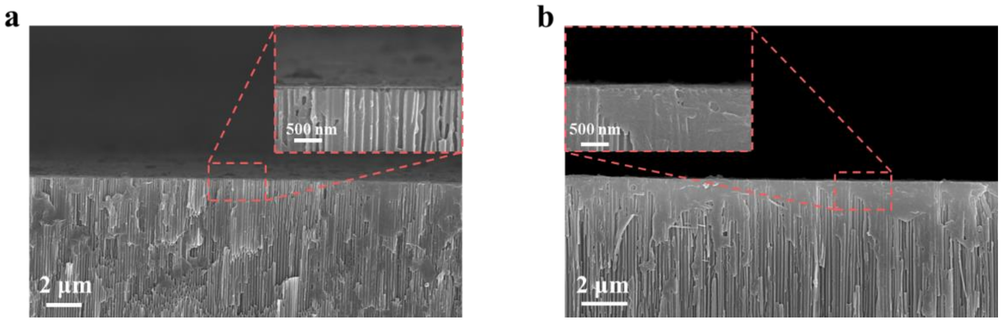

Figure S6. (a, b) Typical cross-sectional SEM images of the assembled monolayer $Ti_3C_2T_x$ and $ZnIn_2S_4$ films on top of the AAO, respectively.

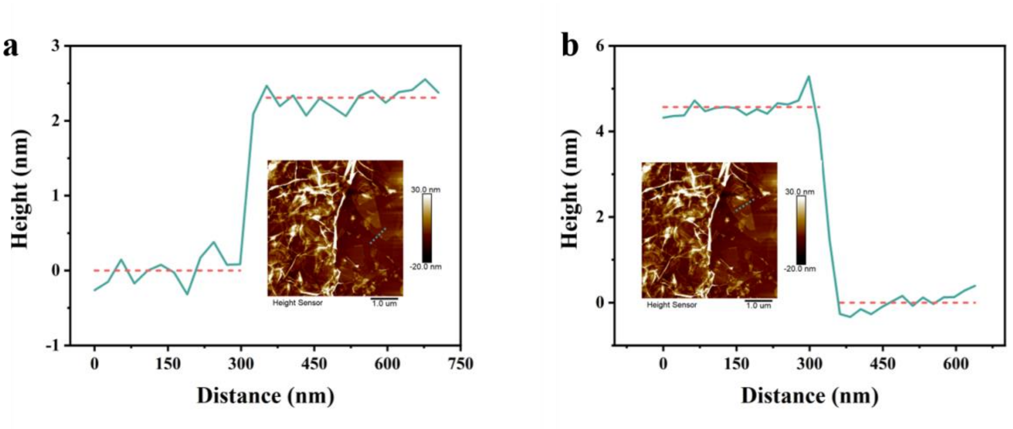

Figure S7. (a, b) Height profiles of the $Ti_3C_2T_x$ films recorded from AFM image in Figure 2c.

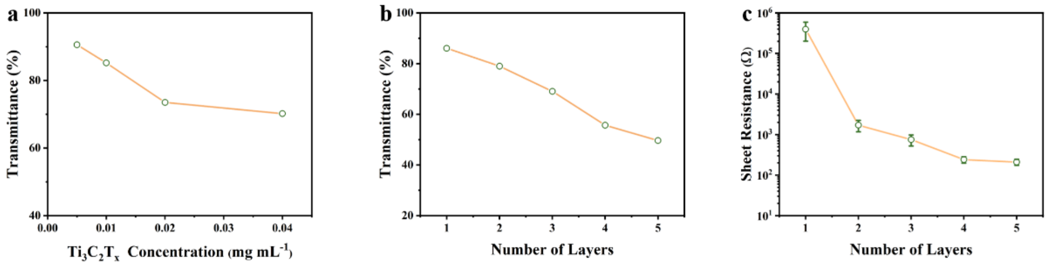

Figure S8. (a) The optical transmittance at 550 nm of the $Ti_3C_2T_x$ films fabricated from solutions with initial different concentrations. (b) The optical transmittance at 550 nm of multilayered $Ti_3C_2T_x$ films. (c) The sheet resistance of the multilayered $Ti_3C_2T_x$ films.

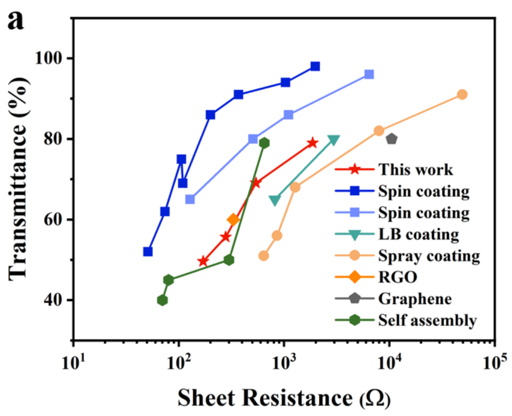

Figure S9. (a) Transmittance at 550 nm as a function of sheet resistance for the $Ti_3C_2T_x$ films and previously reported (data transparent) electrode; RGO: reduced graphene oxide, LB: Langmuir–Blodgett.

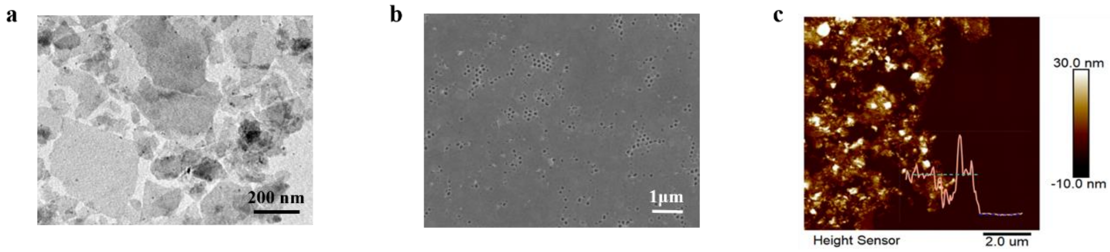

Figure S10. (a) Typical TEM image, (b) SEM image, and (c) AFM image and line-scan height analysis of the assembled monolayer $ZnIn_2S_4$ films, respectively.

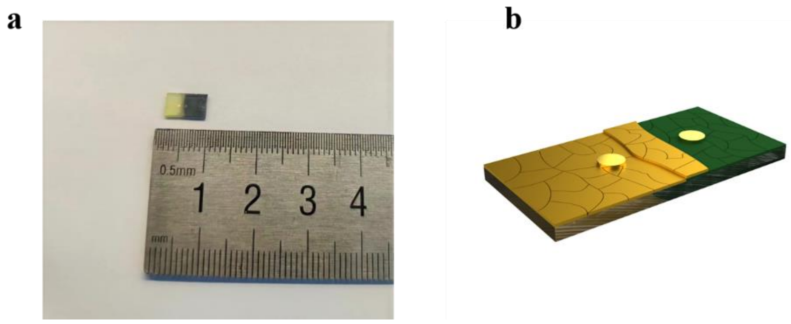

Figure S11. (a, b) The optical image and schematic illustration of the $Ti_3C_2T_x/ZnIn_2S_4$ heterojunction photodetector.

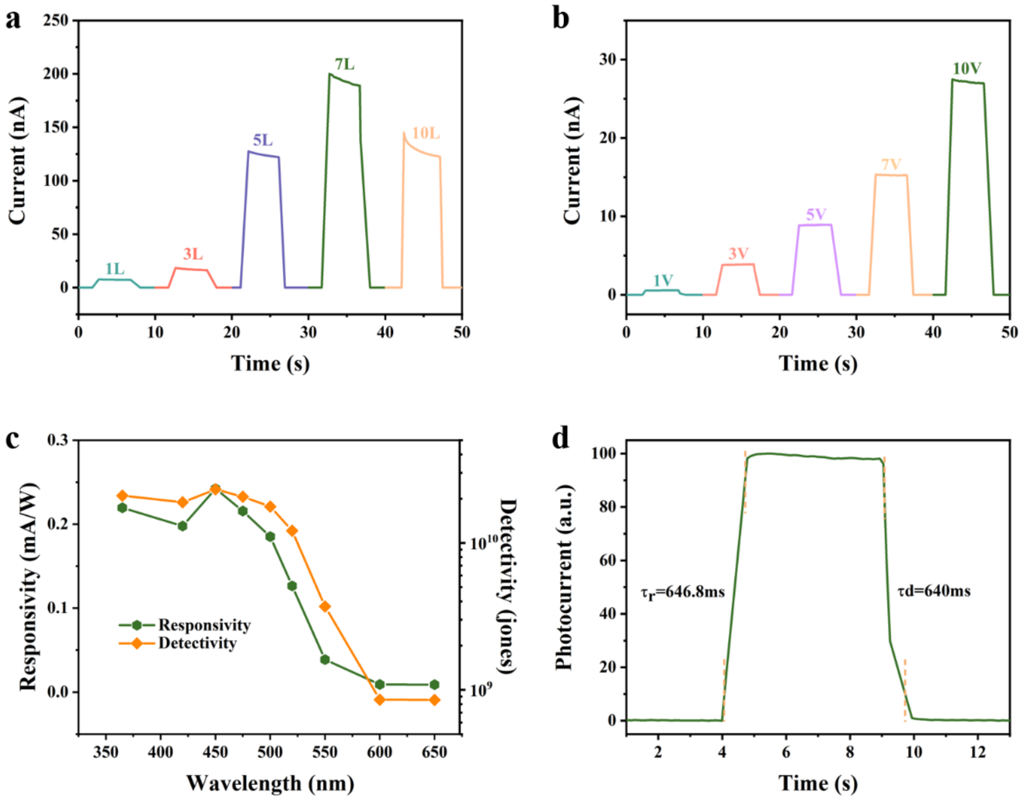

Figure S12. (a) Current–time curves of the $Ti_3C_2T_X$/ZIS heterojunction photodetector with the different number of deposited $ZnIn_2S_4$ films at 10 V bias under a 450 nm light illumination. (b) Current–time curves of the $Ti_3C_2T_X$/ZIS heterojunction photodetector with a different bias voltage under a 450 nm light illumination. (c) Specific detectivity and responsivity of the $Ti_3C_2T_X$/ZIS heterojunction photodetector under illumination with different wavelengths. (d) Response and recovery time of the $Ti_3C_2T_X$/ZIS photodetector.

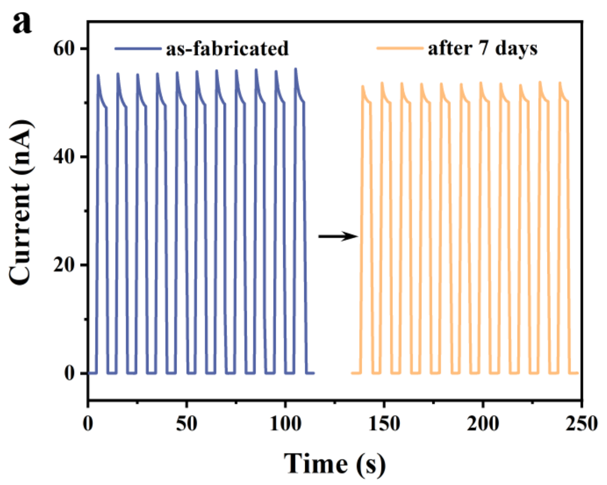

Figure 13. Photo-switching response curves of a $Ti_3C_2T_X$/ZIS photodetector after fabrication and after storage in the ambient environment for 7 days.

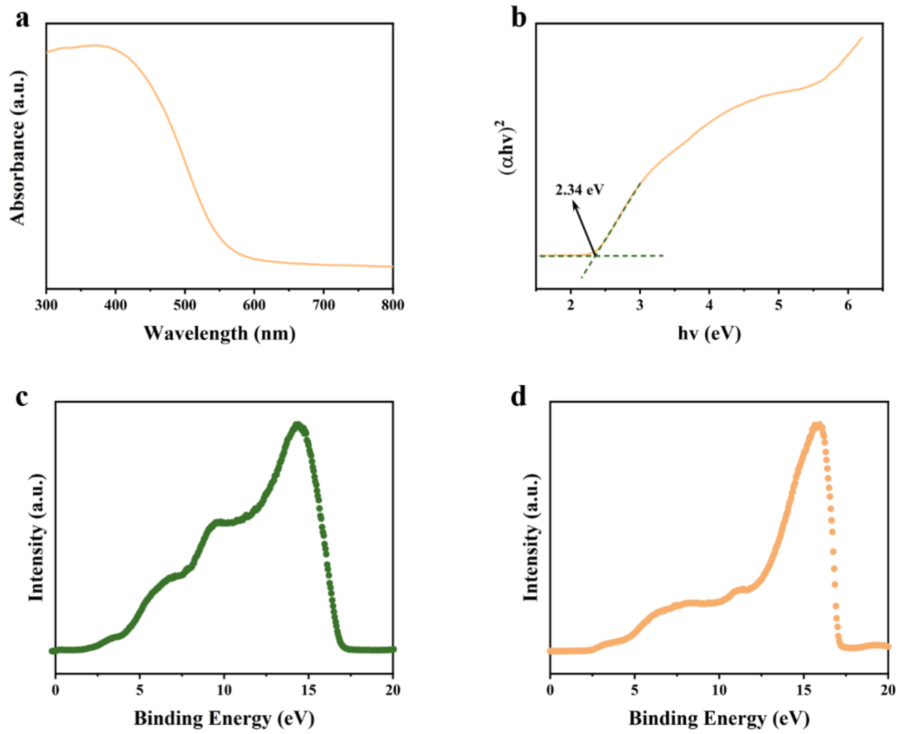

Figure S14. (a) UV/Vis DRS spectrum of $ZnIn_2S_4$ nanoflakes. (b) Band gap estimation by the related curve of $(\alpha h\nu)^2$ versus photon energy (hν) plotted. (c, d) The UPS spectra of $Ti_3C_2T_x$ and $ZnIn_2S_4$ nanoflakes, respectively.

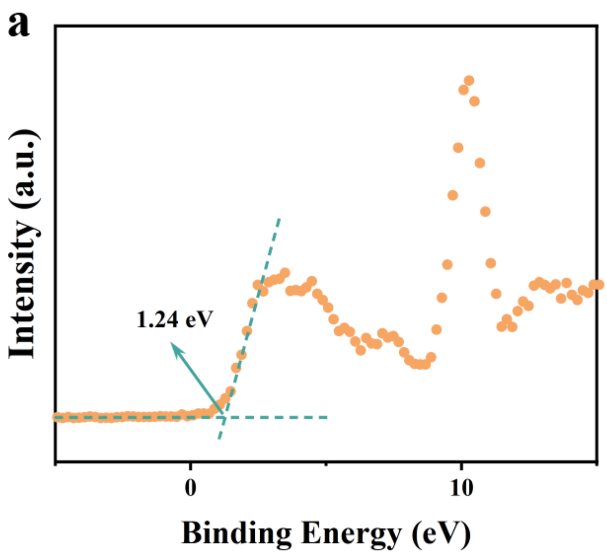

Figure S15. (a) Valence-band (VB) XPS spectrum of the $ZnIn_2S_4$ nanoflakes.

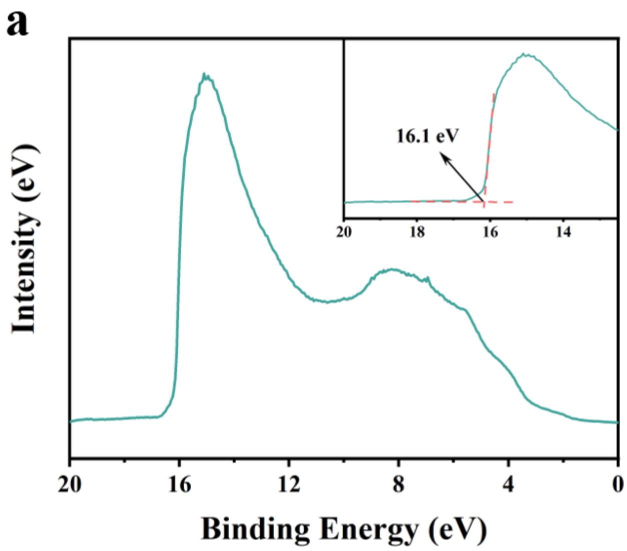

Figure S16. The UPS spectra of Au pad electrodes.
